# Effect of Etching Mode on the Dentin Bond Durability of Universal Adhesives

**DOI:** 10.3290/j.jad.c_2447

**Published:** 2025-12-19

**Authors:** Sena Balaban, Hacer Deniz Arısu

**Affiliations:** a Sena Balaban Research Assistant, Gazi University Faculty of Dentistry, Department of Restorative Dentistry, Biskek St. 1. St. Number: 4 Cankaya, Ankara, Turkey. Conception and design, material preparation, data collection, and analysis, drafted the initial version of the manuscript, critically revised and approved the final manuscript.; b Hacer Deniz Arisu Professor, Gazi University Faculty of Dentistry, Department of Restorative Dentistry, Biskek St. 1. St. Number: 4 Cankaya, Ankara, Turkey. Conception and design, material preparation, data collection, and analysis, critically revised and approved the final manuscript.

**Keywords:** acid etching, dentin-bonding agents, dental bonding, pH

## Abstract

**Purpose:**

This study evaluated the effects of etching mode, pH, and composition on the shear bond strength of three universal adhesives (UAs), in comparison with conventional adhesives.

**Materials and Methods:**

Crowns from 110 extracted third molars were sectioned to obtain 220 dentin surfaces and allocated into 11 groups (n = 20) based on adhesive system (OptiBondFL, ClearfilSE Bond, All-Bond Universal, OptiBond Universal, G2-Bond Universal) and etching mode (etch-and-rinse [ER], self-etch [SE], selective dentin etching [SDE]). Each group was divided into pre- and post-aging sub-groups. Shear bond strength was tested at 24h and after 5,000 thermal cycles. The resin-dentin interface was examined using scanning electron microscopy (SEM). Data were analyzed using one-way ANOVA, LSD post-hoc and paired-sample t-tests (α = 0.05).

**Results:**

All-Bond Universal exhibited the highest bond strength across all modes, while OptiBond Universal showed the lowest. No significant difference was observed between ER and SE modes for most UAs, except G2-Bond Universal. SDE did not result in significantly higher bond strength compared to SE or ER in any group. After aging, G2-Bond Universal in SE mode exhibited the highest bond strength.

**Conclusion:**

Bond strength was influenced by etching mode, pH, and composition of the adhesive. UAs performed comparably to the gold-standard SE adhesive in SE mode; however, their performance in ER mode varied depending on their composition and pH.

**Clinical Relevance:**

To ensure predictable clinical outcomes, clinicians should recognize that UAs do not perform uniformly. Selecting both the adhesive and etching mode according to the adhesive’s composition may enhance long-term bonding success.

The long-term success of adhesive restorations largely depends on the quality of the bond between adhesive systems and dental hard tissues. Effective bonding ensures marginal adaptation, enhances the longevity of restoration, and reduces the risk of secondary caries.^4^ In recent years, universal adhesives (UAs) have gained popularity due to their simplified application protocols, low technique sensitivity, and rapid application.^14^ These adhesives are designed for use in self-etch (SE), etch-and-rinse (ER), or selective-etch modes due to the incorporation of functional acidic monomers in their formulation.^2^

Although phosphoric acid etching provides reliable bonding to enamel, achieving durable adhesion to dentin remains challenging due to its heterogeneous structure, high organic content, and lower mineral content.^3^ The demineralization effect of phosphoric acid is time-dependent; the conventional 15-s etching of dentin may result in over-demineralization, compromising the hybrid layer by exceeding the resin infiltration capacity.^31^ Such over-etching also removes hydroxyapatite from the collagen matrix, which is crucial for chemical bonding with functional monomers. In contrast, the SE mode preserves hydroxyapatite, facilitating more stable bonding.^8,43^ Therefore, alternative modifications to conventional etching protocols have been proposed to overcome their limitations.

Recent studies have investigated the effects of reducing etching time on the bonding performance of UAs.^7,16,17,25,26,34,36^ Stape et al reported that a 3-s application of phosphoric acid may enhance bond strength without over-exposing demineralized collagen.^34^ This technique, known as selective dentin etching (SDE) or short dentin etching, is a relatively novel approach that improves resin–dentin bonding by preserving hydroxyapatite crystals within the intrafibrillar collagen spaces.^5,34^

In addition to etching modes, the chemical formulation and acidity of UAs significantly influence their bonding performance.^8^ UAs exhibit considerable variability in pH, functional monomer, and solvent type. Functional monomers such as 10-methacryloyloxydecyl dihydrogen phosphate (10-MDP), 2-hydroxyethyl methacrylate (HEMA), and glycero-phosphate dimethacrylate (GPDM) modulate the adhesive’s hydrophilic-hydrophobic balance and its interaction with dentin, ultimately affecting bonding performance.^5,41^ Similarly, solvents such as ethanol, acetone, and water differ in their volatility and moisture affinity, influencing monomer infiltration into dentinal tubules and the integrity of a hybrid layer.^41^

In the SE adhesive systems, bonding performance is influenced by the demineralization depth, which is determined by the adhesive’s pH. Strong adhesives produce deeper demineralization, while mild or ultra-mild adhesives result in superficial demineralization, which has been associated with more durable bonding interfaces.^42^ While this relationship is well-established for conventional SE systems, its relevance to UAs remains unclear. Although pH appears to influence the etching mode for UAs,^8,33^ few studies have systematically investigated how pH interacts with adhesive composition and etching modes. In contrast to prior studies that primarily focused on conventional SE and ER modes, the present study evaluates the combined influence of etching mode, adhesive composition, and pH – particularly by incorporating the less-investigated SDE technique – on dentin bond strength

Therefore, this *in vitro* study aimed to evaluate the shear bond strength to dentin of three UAs with varying pH and compositions at different etching modes (ER, SDE, SE) in comparison with conventional SE (two-stage) and ER (three-stage) adhesive systems, which are widely accepted as “gold standard.” The findings of this study may contribute to identifying optimal application strategies for UAs that enhance clinical performance. The following null hypotheses were tested: (i) the etching modes; (ii) the adhesive composition; (iii) pH level would not significantly influence the shear bond strength to dentin.

## MATERIALS AND METHODS

This study was approved by the institutional ethics committee, and informed consent was obtained from all individuals whose teeth, extracted for clinical reasons, were used for research purposes. This study was supported by the institutional scientific research fund.

### Power Analysis

The total sample size was determined to be 220 (20 specimens per group) using the G*Power software package (G*Power Ver. 3.0, Germany) (11, 12) as Type I error rate (α) was set at 0.05, the statistical power (1 – β) was set at 0.80 and the effect size (f) was taken as 0.40 suggested by Cohen (23).

### Materials

The materials used in this study, along with their compositions and manufacturers’ instructions, are presented in Table 1. The adhesives were selected based on the pH classification described by Cuaves-Suarez et al^8^ (ultra-mild, pH ≥ 2.5; mild, pH ≈ 2; intermediately strong, pH ≈ 1.5).

**Table 1:** Materials used in the study, their compositions, and manufacturers’ instructions

Manufacturer(LOT number)	Material	Chemical compositions	pH	Manufacturers’ instructions
Kerr gel etchant Kerr Corporation, Orange, CA, USA(9331345)	Demineralization material	37.5% phosphoric acid	1	Apply acid etchant to dentin surface for 15 s. Rinse thoroughly for 15 s and air-drying for 3 s.
Optibond FL (OPFL), Kerr Corporation, Orange, CA, USA(9331345)	Three-step etch-and-rinse adhesive	Primer: HEMA*, PAMM*, GPDM*, water, ethanol, photoinitiator Adhesive: TEGDMA*, UDMA*, GPDM*, HEMA*, Bis-GMA*, alkali fluorosilicates, photoinitiator	2	Apply primer with scrubbing for 15 s. Air dry for 5 s. Adhesive with scrubbing for 15 s. Air thin for 3 s. Light cure for 10 s.
Clearfil SE (CSE), Kuraray Medical, Okayama, Japan (000461)	Two-step self-etch adhesive	Primer: 10-MDP*, HEMA*, Hydrophilic Dimethacrylate, camphorquinone, N,N-diethanol-p-toluidine, water Adhesive: 10-MDP*, Bis-GMA*, HEMA*, hydrophobic dimethacrylate,camphorquinone, N,N-diethanol-p-toluidine, silanated collodial silica	<2.5	Apply primer and wait for 20 s. Dry thoroughly with mild air flow. Apply adhesive and air flow gently. Light-cure for 10 s.
AllBond Universal (AL), Bisco, Schaumburg, IL, USA (2200004069)	Universal Adhesive	MDP*, Bis-GMA*, HEMA*, ethanol, water, initiator	3.2	Apply two separate coats of adhesive with scrubbing for 10–15 s per coat. Do not light cure between coats. Evaporate excess solvent by thoroughly air-drying for at least 10 s. Light cure for 10 s.
Optibond Universal (OP), Kerr Corporation, Orange, CA, USA (8772891)	Universal Adhesive	GPDM*, HEMA*, ethanol, acetone	2.4	Apply adhesive with scrubbing for 20 s. Dry the adhesive with gentle air first then medium air for 5 s. Light cure for 10 s.
G2 Bond Universal (G2), GC Corporation, Tokyo, Japan) (2208011)	Two-step Universal Adhesive	Primer: 4-MET*, 10-MDP*, 10-MDTP*, acetone, water dimethacrylate monomer, initiator, filler Adhesive: Dimethacrylate monomer, Bis-GMA*, filler, initiator	1.5	Apply primer and wait 10 s. Dry for 5 s with maximum air pressure. Apply adhesive and air-dry for 5 s gently. Light cure for 10 s.
Filtek Z550, 3M ESPE, St. Paul, MN, USA (9457512)	Nanohybrid composite resin	Bis-GMA*, UDMA*, Bis-EMA*, PEGMA*, TEGMA*, 78.5% filler		Apply two layers in 2 mm. Light cure for 20 s per coat.
*Abbreviations: HEMA: 2-Hydroxyethylmethacrylate, PAMM: Phthalic acid monoethyl methacrylate, GPDM: Glycerol Phosphate Dimethacrylate, TEGDMA: Triethylene glycol dimethacrylate, UDMA: Urethane dimethacrylate, Bis-GMA: Bisphenol-A-glycidyl methacrylate, 10-MDP: 10-Methacryloyloxydecyl dihydrogen phosphate, 4-MET: 4-methacryloyloxyethyl trimellitate, 10-MDTP:10-methacryloyloxydecyl dihydrogen thiophosphate, Bis-EMA: Bisphenol-A-ethoxylated-glycidyl dimethacrylate, PEGMA: Polyethylene glycol monomethacrylate, TEGMA: Triethylene glycol dimethacrylate.

### Sample Preparation

**Fig 1:**
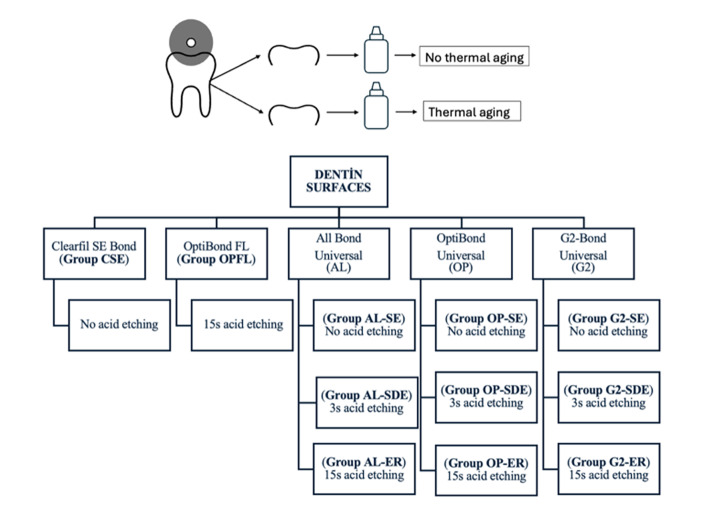
Schematic representation of the study design. The diagram illustrates the classification of specimens into groups based on adhesive systems, etching modes, and thermal aging.

To evaluate the bond strength to dentin, 110 impacted human third molars extracted for orthodontic reasons were used. The teeth were obtained from the outpatient clinic of the Department of Oral and Maxillofacial Surgery, after receiving informed patient consent. The crowns of the teeth were sectioned mesiodistally using a diamond disc with water-cooling, obtaining a total of 220 dentin surfaces. The two sections derived from the same tooth were utilized for pre- and post-aging evaluations within the same group. The dentin surfaces were embedded in cylindrical molds filled with acrylic resin. To form a standard smear layer, the dentin surfaces were ground flat by a polishing machine (Presi Meccapol P230, Grenoble, France) using 600-grit silicon carbide paper under water-cooling at 300 rpm for 60s. The samples were divided into 11 groups (n = 20) according to the adhesive systems (OptiBond FL, Clearfil SE, All-Bond Universal, OptiBond Universal, G2-Bond Universal) and the etching modes (ER, SDE, SE). Groups and surface treatments are presented in Figure 1. All bonding procedures were performed according to the respective manufacturers’ instructions (Table 1).

In the etching groups, the dentin surfaces were etched for either 15s or 3s etching, then rinsed with water for 15s and gently air-dried for 5s to keep the dentin moist. Following surface treatment and adhesive application, an Ultradent jig (Ultradent Products, South Jordan, UT, USA) with a 2.38 mm diameter and 3 mm depth was positioned on the dentin surface, and Filtek Z550 composite resin (3M ESPE, St. Paul, MN, USA) was applied in two layers. Each layer was light-cured using a light-emitting diode curing unit (Valo X, Ultradent Products, UT, USA) in standard mode with an intensity of 1350 mW/cm^2^ for 20s. The light-curing unit was connected to the socket to ensure that the battery level does not affect the light intensity. All samples were stored in distilled water at 37°C for 24 h. Then, half of the samples were artificially aged in a thermal cycler (SD Mechatronic Thermocycler, Feldkirchen-Westerham, Germany) of 5,000 cycles with a dwell time of 30 s and transfer time of 5 s in water baths at 5°C and 55°C, corresponding to 6 months of clinical aging.^43^

### Shear Bond Strength Test

The shear bond strength was measured with a universal testing machine (Schimadzu IG-IS; Kyoto, Japan). The load was applied to the dentin-composite interface using a knife-edge blade at a cross-head speed of 1 mm/min until fracture. The maximum load (Newton) was converted to megapascal (Mpa) based on the bonding area.

### Failure Mode Analyses

The fractured surfaces were analyzed using a stereomicroscope (Olympus SZ2-LGB, Tokyo, Japan) (×40) and categorized as adhesive (when failure occurs at the adhesive interface), cohesive (failure occurs within the dentin or resin), and mixed (failure occurs at the adhesive interface and leaves adhesive or resin residue on the dentin surface).^35^

### Scanning Electron Microscopy Analysis

Two additional samples for each group were prepared for scanning electron microscopy (SEM) analysis. Standardized Class I cavities were prepared with the cavity floor parallel to the occlusal surface, restored with adhesive and composite resin, and stored in distilled water for 24 h. Afterwards, the crowns were sectioned mesiodistally to obtain cross-sections for SEM evaluation. The specimens were treated with 37% orthophosphoric acid for 5 s, rinsed, and immersed in 5% NaOCl for 10 min.^10^ All samples were gold-sputtered and analyzed under SEM (Hitachi SU5000; Japan).

### Statistical Analysis

Statistical analysis was conducted using IBM SPSS 25.0 (SPSS, Chicago, IL, USA). Data normality was assessed using Kolmogorov–Smirnov and Shapiro–Wilk tests. Homogeneity of variance was evaluated with Levene’s test. One-way ANOVA and LSD post-hoc test were used for group comparisons. A paired-sample t-test was performed to compare the immediate and aged subgroups. The significance level was determined as α = 0.05.

## RESULTS

### Shear Bond Strength Test Results

The mean and standard deviations of the immediate and aged groups, along with statistical comparisons, are presented in Table 2.

**Table 2:** Shear bond strength values of different adhesive groups at immediate and thermal aging

	Shear bond strength (Mean ± Sd)	Paired sample t-test (p)
Immediate	Thermal aging
CSE	17.71 + 7.48 ^cdeA^	11.25 ± 5.09 ^B^	0.033*
OPFL	18.96 ± 9.97 ^deA^	14.39 ± 7.32 ^A^	0.359
AL-SE	20.96 ± 4.22 ^eA^	14.24 ± 8.91 ^A^	0.109
AL-SDe	14.77 ± 7.03 ^bcdA^	13.39 ± 4.19 ^A^	0.493
AL-ER	16.91 ± 6.4 ^cdeA^	15.79 ± 6.98 ^A^	0.754
OP-SE	12.23 ± 7.36 ^abcA^	12.24 ± 6.79 ^A^	0.998
OP-SDe	11.1 ± 5.69 ^abA^	12.97 ± 5.97 ^A^	0.486
OP-ER	10.51 ± 5.77 ^abA^	8.13 ± 3.65 ^A^	0.408
G2-SE	14.9 ± 4.98 ^bcdA^	16.33 ± 8.72 ^A^	0.609
G2-SDe	12.09 ± 5.33 ^abcA^	11.69 ± 4.9 ^A^	0.866
G2-ER	7.87 ± 4.45 ^aB^	12.24 ± 3.82 ^A^	0.005*
ANOVA (P =)	< 0.001*	0.057	
F	3.815	1.864	
η^2^	0.276	0.157	
Different lower-case letters in the same column indicate the difference in shear bond strength between the groups according to the LSD test result. Different capital letters in the same row indicate the statistical difference in the shear bond strength of the same adhesive before and after thermal aging (A>B) according to the paired sample t-test result. Asterisks (*) indicate statistically significant differences (P < 0.05).

The highest immediate shear bond strength was observed in the AL-SE group, while the lowest was recorded in the G2-ER group. All-Bond Universal exhibited the highest bond strength across all etching modes. For All-Bond Universal (AL), ER and SE modes showed comparable results (P > 0.05). In contrast, SDE demonstrated significantly lower bond strength compared with SE (P < 0.05) and also tended to be lower than ER, although this difference was not statistically significant. For OptiBond Universal (OP), no significant differences were observed among the three etching modes (P > 0.05). For G2-Bond Universal (G2), ER mode yielded the lowest bond strength values, which were significantly lower than SE (P < 0.05). The SDE group showed no significant difference compared with either SE or ER (P > 0.05). UAs in SE mode showed comparable bond strength to Clearfil SE Bond (P > 0.05), whereas their ER mode bond strengths were generally lower than OptiBond FL, except for AL-ER (P > 0.05).

After thermal aging, the G2-SE group showed the highest bond strength, while the OP-ER group demonstrated the lowest. A significant decrease in bond strength was observed in the CSE (Clearfil SE) group after aging, whereas G2-ER exhibited a significant increase (P < 0.05). Other groups showed no statistically significant differences between immediate and aged results.

### Failure Mode Analysis Results

**Fig 2:**
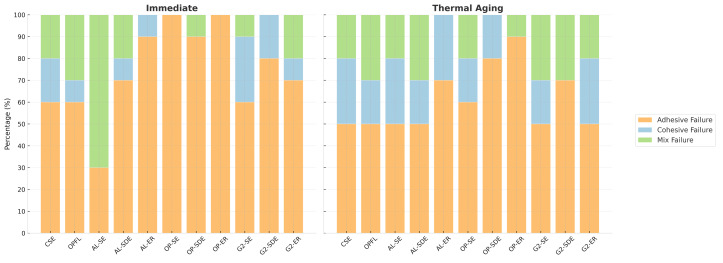
Distribution of failure modes (adhesive, cohesive, mixed) among the test groups.

Results from fracture mode analysis are shown in Figure 2. Failure mode analysis revealed predominantly adhesive failures in all groups, except for the AL-SE group, where mixed failures were more common (Fig 2)

### SEM Analysis

**Fig 3:**
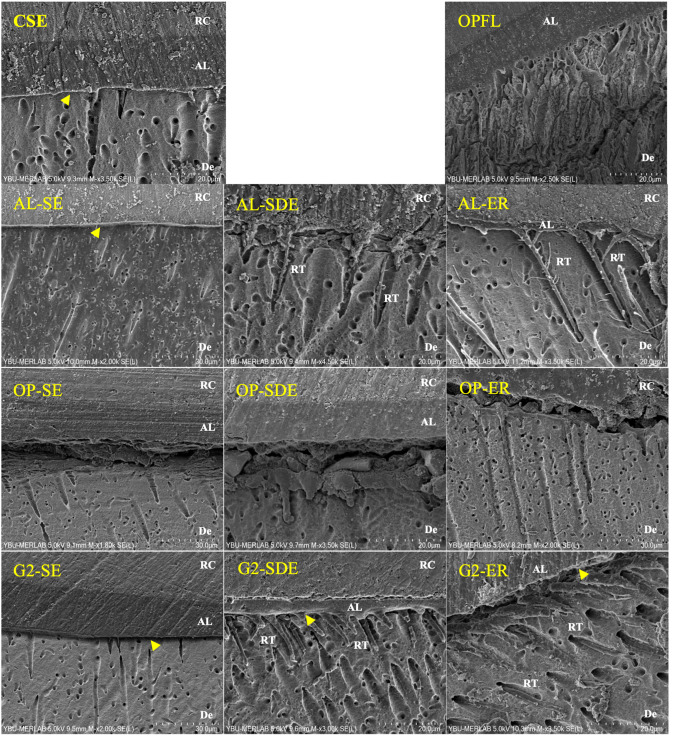
Representative SEM images of resin–dentin interfaces for different etching modes: ER: Etch-and-rinse, SDE: Selective Dentin Etching, SE: Self-etch (Magnifications: ×20,000 and ×30,000 as indicated in each image). The hybrid layer is indicated by yellow arrows. RT: Resin tag AL: Adhesive layer De: Dentin RC: composite resin.

SEM images of bonding interfaces are presented in Figure 3. The morphological characteristics of the interfaces exhibited notable variations depending on the etching mode. In the OptiBond Universal group, partial interfacial separation was observed at the bonding interface. A continuous and homogeneous hybrid layer was visible in the SE mode, which appeared more distinct in the G2-Bond Universal group. Additionally, in the ER mode, numerous long resin tags were observed; in the SDE mode, resin tags were fewer and shorter; and in the SE mode, they appeared thin and sparse.

## DISCUSSION

Although UAs are introduced as multi-mode adhesives, the optimal etching strategy for dentin bonding remains unclear. Meta-analyses suggest that ER mode may enhance bond strength in ultra-mild and intermediately strong UAs, whereas mild UAs show comparable performance across etching modes.^8,33^ However, clinical studies have reported that different etching modes do not significantly influence long-term bonding outcomes.^27,28^ More recently, SDE has been proposed to improve bonding by limiting collagen overexposure^7,17,34^; however, supporting clinical evidence remains limited. In the present study, all adhesives – except G2-Bond Universal – demonstrated similar bond strength in SE and ER modes, and SDE did not provide statistically or clinically superior performance. Taken together, these findings indicate that while most adhesives performed similarly regardless of etching mode, G2-Bond Universal showed distinct behavior, resulting in the partial rejection of the first null hypothesis.

Stape et al^34^ investigated the effect of etching times on the Ca ratio and bond strength to dentin and reported that a 3-s phosphoric acid application resulted in a higher bond strength without altering Ca ratio compared to SE mode. In contrast, in the present study, following 3 s acid application (SDE) the shear bond strength of adhesives was comparable to or lower than that observed in the SE mode. This discrepancy may be attributed to the adhesive used in Stape et al’s study, which contains polyalkenoic acid – a component capable of chemically bonding with hydroxyapatite in addition to 10-MDP.^19,32^ However, the Ca ratios were not calculated in our study. Future studies should investigate the effects of adhesives with different pH levels on bond strength and Ca ratio in dentin.

When comparing UAs with adhesives considered to be gold standards, All-Bond Universal demonstrated similar bond strength to OptiBond FL and Clearfil SE Bond, consistent with the Jang et al.^22^ In contrast, the other adhesives tested in this study exhibited significantly lower bond strength in ER mode compared to OptiBond FL, while yielding similar bond strength to Clearfil SE Bond in SE mode. The comparable bond strength observed among all UAs in SE mode and Clearfil SE Bond may be attributed to the presence of a self-etch component within the formulation of universal adhesives. Similar to Clearfil SE Bond, these systems combine acidic monomers and primer agents in a single bottle, allowing smear layer modification without excessive dentin demineralization. This shared mechanism may account for the comparable results observed.^9^

Among the tested UAs, G2-Bond Universal has the lowest pH and demonstrated the lowest immediate bond strength when applied in ER mode. The combination of acid application and the inherently low pH of G2-Bond Universal may have further increased the acidity of the medium, thereby negatively affecting the initial polymerization reaction of the adhesive. Previous studies have reported that in adhesive systems employing the commonly used camphorquinone/amine initiator system, amine co-initiators can be protonated and deactivated in low pH environments.^15,30^ Therefore, the lowest bond strength of G2-Bond Universal in ER mode may be related to insufficient polymerization under acidic conditions. However, the manufacturer does not disclose the exact composition of the initiator system in G2-Bond Universal. To draw more definitive conclusions, further information from the manufacturer or additional investigations would be necessary to clarify the exact mechanism involved.

Katsuki et al^24^ reported that the shear bond strength of G2-Bond Universal in both ER and SE modes increased after thermal aging, which they attributed to a possible ongoing polymerization reaction after light curing. In the present study, a significant increase after thermal aging was observed only in the ER mode, while no significant changes occurred in SE or SDE. Therefore, although our findings partially support the observations of Katsuki et al, the underlying mechanism – such as a potential post-polymerization reaction – remains uncertain.

HEMA content also influenced bonding performance. HEMA is a hydrophilic monomer that retains water within the adhesive layer, making complete removal during air-drying more difficult; consequently, residual water in the adhesive layer may impair polymerization efficiency.^30^ In contrast, studies have shown that HEMA-free adhesive systems result in adhesive layers that are more hydrophobic and resistant to hydrolytic degradation.^1,4,21,24,37,39,40^ In the present study, although thermal aging did not yield statistically significant differences, the highest bond strength was observed in the HEMA-free G2-Bond Universal applied in SE mode. Consistent with these observations, Hanabusa et al reported that the bonding interface of HEMA-free UA is more prone to degradation in ER mode than in SE mode.^14^ Similarly, in the present study, G2-Bond Universal demonstrated higher bond strength in SE mode than in ER mode after thermal aging, although the difference was not statistically significant.

Furthermore, certain application factors may have influenced the bonding performance observed in this study. Huang et al^18^ reported that implementing a 10-s waiting period following the adhesive application improves dentin bond strength by promoting a thicker and more uniform hybrid layer and enhances adequate dentin demineralization. In the present study, only G2-Bond Universal was applied with a waiting time of 10 s, in accordance with the manufacturer’s instructions. SEM images revealed a continuous and homogeneous hybrid layer when G2-Bond Universal was applied in SE mode. A homogeneous hybrid layer provides uniform stress distribution at the bonding interface, which contributes to high bond strength.^36^ In light of the findings of Huang et al., the use higher-pH adhesives without a waiting period in this study might have contributed to insufficient dentin demineralization. Future studies should investigate the effects of waiting times on bond strength and determine the optimal waiting period for other adhesives as well.

Solvents such as acetone and ethanol in adhesives are responsible for the penetration of the adhesives into the collagen network by displacing water.^20^ Because of its low hydrogen bonding capacity, it cannot re-expand to shrunken collagen; hence, the ‘wet bonding technique’ is recommended for acetone-based adhesive systems. The major disadvantage of acetone-based adhesives is their high moisture sensitivity, whereas ethanol-based adhesives are less sensitive.^20,29,41^ The high moisture sensitivity of acetone-based adhesives could explain their low shear bond strength, particularly in ER mode. Particularly in the ER mode, the bond strength of the ethanol-based adhesive All-Bond Universal was higher than that of the acetone-based adhesives G2-Bond Universal and OptiBond Universal, consistent with previous studies.^6,29^

Fujiwara et al^13^ reported that double-layer adhesive application improves dentin bond strength by enhancing monomer infiltration, promoting solvent evaporation, and producing a more uniform adhesive layer. However, other studies have demonstrated that this technique does not affect the bond strength of adhesives containing fillers.^13,38^ Therefore, understanding the composition of the adhesive is crucial when selecting the appropriate application method. Among the adhesives tested, All-Bond Universal was the only one applied in the double-layer technique on dentin. This may be one possible reason why it showed comparable or superior bond strength across all etching modes and aging conditions.

Differences in functional monomers also contributed to variations in bonding effectiveness. GPDM, the functional monomer of OptiBond Universal, is more hydrophilic than 10-MDP due to its shorter carbon chain.^37,45^ Wang et al^45^ reported that GPDM promotes demineralization of the dentin surface instead of forming stable bonds with calcium in hydroxyapatite. This leads to the formation of unstable calcium phosphate salts within the hybrid layer. In contrast, 10-MDP forms a strong and stable chemical bond with calcium in hydroxyapatite, which contributes to higher bond strength.^37,45^ Among the UAs tested in the present study, OptiBond Universal was the only adhesive containing GPDM, which demonstrated the lowest bond strength.

Statistical analysis revealed significant differences in bond strength among adhesives with different chemical compositions and pH (Table 2, P < 0.05). Accordingly, the second and third null hypotheses – stating that adhesive composition and pH have no effect on bond strength – were rejected. However, since pH and chemical composition are interdependent properties of UAs, their influence on dentin bonding effectiveness should be interpreted collectively as characteristics of the adhesive system, rather than as independent variables.

Previous systematic reviews and meta-analyses have demonstrated that the bond strength of UAs to dentin is influenced by their pH.^8,33^ In accordance with the findings of Wagner et al,^44^ our study showed that the ultra-mild adhesive (All-Bond Universal) exhibited the highest immediate shear bond strength across all etching modes. The lowest shear bond strength value was obtained from the mild adhesive (OptiBond Universal). In contrast to our findings, the systematic review by Cuaves-Suarez et al reported higher bond strength values with mild UAs.^8^ It should be noted, though, that the adhesives included in that review were primarily 10-MDP–containing mild UAs, which differ from those evaluated in the present study. Nevertheless, considering the discrepancy between the findings of the systematic review and our results, it may be suggested that while both composition and pH can influence the bond strength of adhesives, compositional differences among UAs appear to play a more decisive role under the conditions tested in this study. However, as a limitation, the simultaneous variation in both pH and chemical composition among the adhesives tested may restrict the possibility of drawing definitive conclusions regarding the relative contribution of each factor.

Although this *in vitro* study employed thermocycling protocols simulating six months of clinical aging, it does not fully account for the multifactorial intraoral conditions that affect the longevity of dental restorations, including oral hygiene, salivary microflora, and occlusal forces. Moreover, the lack of significant differences between immediate and aged bond strengths in many groups suggests that the 6-month thermal cycling protocol may be insufficient to fully simulate clinical aging. Future studies should consider longer thermal cycling durations and employ aging methods that more accurately replicate the intraoral environment. An additional limitation is the variation in both chemical composition and pH among the adhesives tested, which may limit the comparability of the results. Future studies utilizing experimental adhesives with similar chemical composition but different pH values – or vice versa – may more effectively clarify the individual effects of pH and chemical formulation on bond strength. In addition, it was not possible to obtain adhesive interfaces perfectly perpendicular to the dentinal tubules in all specimens, which may have affected the clarity of some SEM images and should be taken into account in the interpretation of interfacial morphology.

## CONCLUSION

Within the limitations of this *in vitro* study, the bonding effectiveness of UAs appeared to be influenced by etching mode as well as their pH and chemical composition. When applied in SE mode, UAs generally showed bond strength values comparable to the gold standard SE adhesive. In contrast, their performance in ER mode seemed more sensitive to differences in chemical composition and pH. The SDE mode did not demonstrate a clear advantage in bond strength under the conditions tested. Overall, these findings suggest that bonding strategies may need to be tailored to the specific chemical characteristics of each adhesive system, although further studies are required to confirm these observations.

### Clinical Relevance

To achieve long-term clinical success with adhesive restorations, clinicians should be aware that UAs may not perform uniformly across all etching modes. Therefore, both the choice of adhesive and the etching mode may need to be tailored to the specific properties of each adhesive. Such an individualized approach could contribute to achieving more durable and predictable outcomes in adhesive restorations.

### Acknowledgments

#### Conflict of interest

The authors declare that there are no conflicts of interest related to the authorship or publication of this manuscript.

#### Funding

This study was supported by the Gazi University Scientific Research Projects with ID code 8535.

#### Ethical approval

This study was approved by the Ethics Board of the Gazi University Faculty of Dentistry (GUDHKAEK.2022.25/2).
